# Prognostic Factors Associated with Acute Heart Failure in Patients Admitted for COVID-19: Analysis of the SEMI-COVID-19 Registry

**DOI:** 10.3390/jcm12144649

**Published:** 2023-07-12

**Authors:** Manuel Méndez Bailón, Noel Lorenzo Villalba, Jorge Garcia Onrubia, Manuel Rubio Rivas, Maria Victoria Nuñez Rodriguez, María de los Reyes Pascual Pérez, Carmen Díaz Pedroche, Eva María Fonseca Aizpuru, Maria Victoria Villalba Garcia, Gema Maria Garcia Garcia, Paula María Pesqueira Fontán, Arturo Artero, Esther Montero Hernandez, José Nicolás Alcalá Pedrajas, Vicente Giner Galvan, Daniel Monge Monge, Laura Letona Giménez, Miriam García Gómez, Carmen Martínez Cilleros, Nuria Puente Ruiz, Joaquin Escobar Sevilla, Raquel Gómez Méndez, José Manuel Ramos-Rincón, Ricardo Gomez Huelgas, on behalf of the SEMI-COVID-19 Network

**Affiliations:** 1 Department of Internal Medicine Hospital Clínico San Carlos, Hospital Clinico San Carlos (IdISSC), 28040 Madrid, Spain; manuelmenba@hotmail.com (M.M.B.);; 2 Department of Internal Medicine, Bellvitge University Hospital, L’Hospitalet de Llobregat, 08907 Barcelona, Spain; 3 Internal Medicine Department, Costa del Sol Hospital, 29603 Marbella, Spain; 4 Hospital General de Elda, 03600 Elda Alicante, Spain; 5 Internal Medicine Department, Hospital Universitario 12 de Octubre, 28041 Madrid, Spain; 6 Department of Internal Medicine, Hospital de Cabueñes, 33394 Gijón, Spain; 7 Hospital Universitario Gregorio Marañón, 28007 Madrid, Spain; 8 Hospital Universitario de Badajoz, 06080 Badajoz, Spain; 9 Department of Internal Medicine, Complejo Hospitalario Universitario de Santiago, Santiago de Compostela, 15706 A Coruña, Spain; 10 Hospital Universitario Dr. Peset, 46017 Valencia, Spain; 11 Department of Internal Medicine, Hospital Universitario Puerta de Hierro—Majadahonda, CIBERCV, Joaquín Rodrigo 1, 28222 Majadahonda, Spain; 12 Hospital de Pozoblanco, 14400 Córdoba, Spain; 13 Hospital Universitari Sant Joan D’Alacant, 03550 Sant Joan d’Alacant, Spain; 14 Internal Medicine Department, Segovia Hospital Complex, 40002 Segovia, Spain; 15 Hospital Universitario Miguel Servet, 50009 Zaragoza, Spain; 16 Internal Medicine Department, Urduliz Alfredo Espinosa Hospital, 48610 Urdúliz, Spain; 17 Internal Medicine Department, HLA Moncloa University Hospital, 28008 Madrid, Spain; 18 Servicio de Medicina Interna, Hospital UM Valdecilla, 39008 Santander, Spain; 19 Internal Medicine Department, Virgen de las Nieves University Hospital, 18014 Granada, Spain; 20 Hospital Universitario Lucus Augusti, 27003 Lugo, Spain; raquel.gomez.mendez@gmail.com; 21 Departamento de Medicina Clínica, Medicine School, University Miguel Hernández, 03020 Elche, Spain; 22 Departmento de Medicina Interna, Instituto de Investigación Sanitaria y Biomédica de Alicante (ISABIAL), Hospital General Universitario Dr. Balmis, 03010 Alicante, Spain; 23 Internal Medicine Department, Regional University Hospital of Málaga, Biomedical Research Institute of Málaga (IBIMA), University of Málaga (UMA), 29016 Málaga, Spain

**Keywords:** prognostic factors, heart failure, COVID-19

## Abstract

Introduction: Since the beginning of the COVID-19 pandemic in March 2020, an intimate relationship between this disease and cardiovascular diseases has been seen. However, few studies assess the development of heart failure during this infection. This study aims to determine the predisposing factors for the development of heart failure (HF) during hospital admission of COVID-19 patients. Methodology: A retrospective and multicenter study of patients with HF admitted for COVID-19 in 150 Spanish hospitals (SEMI-COVID-19 Registry). A bivariate analysis was performed to relate the different variables evaluated in patients developing heart failure during hospital admission. A multivariate analysis including the most relevant clinical variables obtained in bivariate analyses to predict the outcome of heart failure was performed. Results: A total of 16.474 patients hospitalized for COVID-19 were included (57.5% men, mean age 67 years), 958 of them (5.8%) developed HF during hospitalization. The risk factors for HF development were: age (odds ratio [OR]): 1.042; confidence interval 95% (CI 95%): 1.035–1.050; *p* < 0.001), atrial fibrillation (OR: 2.022; CI 95%: 1.697–2.410; *p* < 0.001), BMI > 30 kg/m^2^ (OR: 1.460 CI 95%: 1.230–1.733; *p* < 0001), and peripheral vascular disease (OR: 1.564; CI 95%: 1.217–2.201; *p* < 0.001). Patients who developed HF had a higher rate of mortality (54.1% vs. 19.1%, *p* < 0.001), intubation rate (OR: 2,36; *p* < 0.001), and ICU admissions (OR: 2.38; *p* < 0001). Conclusions: Patients who presented a higher risk of developing HF were older with cardiovascular risk factors. The risk factors for HF development were age, atrial fibrillation, obesity, and peripheral vascular disease. In addition, patients who developed HF more frequently required to be intubated or admitted to the ICU.

## 1. Introduction

Since the first cases of COVID-19 emerged in March 2020, an intimate relationship has been seen between this disease and cardiovascular disease. Although some links among these conditions have been described, they have not been fully elucidated. Furthermore, the study of this relationship may be hampered by the overlapping symptomatology of COVID-19 infection with other cardiac diseases such as heart failure (HF) and coronary heart disease.

The relationship between COVID-19 and cardiovascular diseases can be reflected in several ways: (1) mortality from COVID-19 is 10.5% higher in those individuals with cardiovascular disease [1], (2) COVID-19 infection can manifest as an acute coronary syndrome, even in individuals without prior cardiac comorbidities, and (3) 14% of individuals suffer acute cardiac damage in the course of COVID-19 infection [2].

COVID-19 infection can either precipitate heart failure in previously healthy individuals or cause a flare in individuals with this previous condition [3]. In several studies, de novo heart failure has been observed in 1 in 4 patients hospitalized with COVID-19 and in 1 in 3 patients admitted to the intensive care unit (ICU) [4,5].

The mechanism by which COVID-19 produces myocardial damage is multiple. Among the different causes of cardiac damage, we can find: (1) those mechanisms shared with other infections such as fever, excess sympathetic activation and tachycardia, which increase oxygen and energy consumption [3,6,7]; (2) cardiac damage produced by the hyperinflammatory response triggered by COVID-19 infection, which can not only trigger acute HF both in patients with or without chronic HF [4,5] but also other complications (thromboembolic phenomena, renal failure, shock) [8,9]; (3) myocardial damage due to direct infection of the myocardium by the ACE-2 receptor-dependent virus. The entry of the virus through this receptor produces a decrease in the number of receptors in the membrane, preventing the cardioprotective effects of this membrane protein [9]; finally, the renal failure described in 15–29% of patients may lead to a volume overload that precipitates the exacerbation of previous HF [8,9]. Upregulation in the heart may allow the SARS-CoV-2 to infect the conduction pathways more easily, which can lead to arrhythmia. QT-prolonging medications, induced proinflammatory state, demand ischemia, myocarditis, or underlying heart conditions can also induce arrhythmias in COVID-19 patients [10]. Endothelial dysfunction is a pathological consequence of COVID-19 contributing to cardiovascular disease and not a cause. Some studies indicated direct infection of ECs by SARS-CoV-2 while others proposed that endothelial dysfunction resulted from an indirect, bystander effect of infection of epithelial cells, activation of neutrophils and platelets, or elevation of proinflammatory cytokines in COVID-19 [11,12]. Dysregulation of the kallikrein-kinin system (KKS) is yet another etiologic mechanism of EC dysfunction in COVID-19 [13]. Investigations suggest that pericytes represent the primary target of SARS-CoV-2 in the heart. Infection of pericytes can account for the observed pericyte and endothelial cell death, innate immune response, and immunothrombosis usually described in COVID-19 hearts [11]. Initial autopsy reports suggested that SARS-CoV-2 myocarditis was common; however, subsequent studies rarely detected myocarditis and described the presence of microthrombi, cardiomyocyte necrosis, and inflammatory infiltrates without cardiomyocyte damage more common [11].

The aim of this study was to compare the clinical characteristics of patients who develop HF during hospital admission for COVID-19 versus those who do not develop HF after COVID-19 infection and to determine the predictor factors for the development of HF during hospital admission for COVID-19.

## 2. Methodology

### 2.1. Study Design

An observational, retrospective, multicenter, nationwide study was conducted on patients hospitalized for coronavirus infection from 1 March to 1 October 2020. Data were extracted from the SEMI-COVID-19 registry developed by the Spanish Society of Internal Medicine (SEMI), in which a total of 150 Spanish hospitals participated [14]. This registry includes patients admitted consecutively for COVID-19 disease, confirmed microbiologically in all cases by reverse transcription polymerase chain reaction (RT-PCR) either by nasal swab, saliva, or bronchoalveolar lavage and/or antigen test. Patients who developed acute HF during hospitalization were compared with those who did not develop acute HF, regardless of whether they had previously been diagnosed with HF, considering this as another risk marker. Factors differing in each group were studied.

### 2.2. Variables

Clinical, epidemiological, radiological, and therapeutic variables were collected and analyzed from the SEMI-COVID-19 registry database during the hospital admission period. The diagnosis of acute HF was based on the diagnostic criteria of the attending physician who attended the patient admitted for COVID-19. Patients were considered to have hypertension, diabetes, or dyslipidemia if there was a previous diagnosis or if they were receiving pharmacological treatment for these pathologies. In the case of diabetes, two subdivisions were made, one for those with target organ damage (cerebral, cardiovascular, renal, or retinal) and another for those with no recognizable damage. Cardiovascular arteriosclerotic disease included acute myocardial infarction, acute coronary syndrome, angina, and previous coronary revascularization. Cancer included all solid tumors with or without metastases excluding non-melanocytic skin tumors. Moderate-severe chronic kidney disease was defined by a glomerular filtration rate < 45 mL/min/1.73 m^2^ according to the CKD-EPI equation and moderate-severe liver failure included classes B and C of the Child-Pugh classification.

Laboratory data (blood count, biochemistry, blood gases, coagulation) and complementary imaging tests such as chest X-ray’s were collected. Treatments were classified into 5 groups: (1) antimicrobial, (2) immunomodulatory therapy, (3) anticoagulants, (4) ventilatory support, and (5) pronation therapy. In-hospital complications included admission to the ICU, acute respiratory distress, acute coronary syndrome, arrhythmia, shock, sepsis, acute renal failure, deep vein thrombosis, or acute arterial ischemia.

### 2.3. Statistical Analysis

Patients were classified into two categories according to the presence or absence of acute HF during hospital admission defined according to clinical criteria. Qualitative and quantitative variables are expressed as absolute values and percentages and as means and ranges, respectively. A bivariate analysis was performed; the chi-square test was used for the analysis of qualitative variables and the Student’s *t*-test for quantitative variables. Logistic regression analysis was performed with the statistically significant variables in the bivariate analysis of epidemiological and comorbidity variables to predict HF as an outcome variable (*p* < 0.05). SPSS 26.0 statistical software was used.

### 2.4. Ethical Aspects

This study was performed within the SEMI-COVID registry and was approved by the Ethics Committee of the Hospital de Málaga for the retrospective analysis of variables and data collection.

## 3. Results

Of the 16,474 patients hospitalized for COVID-19 included in the study, 958 (5.8%) developed an episode of HF as a complication during hospital admission. The different epidemiological, clinical, analytical, and treatment factors related to the development of this complication during admission are shown in Table 1, Table 2, Table 3, Table 4 and Table 5.

Those patients who developed HF were significantly older, obese, had more cardiovascular risk factors, and had more history of cardiovascular disease. They also significantly had other important non-cardiovascular diseases, such as chronic obstructive pulmonary disease (COPD), HIV infection, dementia, or solid cancer without metastasis (Table 1).

Regarding symptoms, the presence of dyspnea, ageusia, anosmia, arthromyalgia, nausea, diarrhea, headache, confusion, and seizures was significantly associated with the development of HF, whereas vomiting, anorexia, asthenia, and pharyngeal discomfort were not significantly different in both groups.

Exploratory signs such as crackles, rhonchi, and wheezing were significantly associated with the development of HF. Those patients who developed HF had lower diastolic blood pressure than those who did not, while no statistically significant differences were observed in systolic blood pressure in both groups (Table 2).

Regarding analytical parameters, those patients who developed HF had worse renal function, lower oxygen saturation, higher CRP, D-dimer, leukocytes, glycemia, potassium, and procalcitonin values, as well as lower pH values, lower platelet count or hypoalbuminemia more frequently (Supplementary Table S1). Interestingly, IL-6 or fibrinogen levels did not vary significantly in both groups.

The use of antibiotics (except for macrolides) and corticosteroids was more frequent in those patients who developed HF. Likewise, the usual treatment with IECAS, ARA II, beta-blockers, and anticoagulants was more frequent among patients who developed this complication (Table 3).

Those patients who developed HF during admission had a higher percentage of complications or required admission to the ICU or intubation (Table 4).

After performing logistic regression analysis, it was observed that the risk factors associated with the development of HF were atrial fibrillation, BMI > 30 kg/), myocardial infarction, and the previous HF (Table 5).

## 4. Discussion

Despite the large number of studies on COVID-19 and risk factors for the development of severe COVID-19 and death from COVID-19, there are still emerging reports about the risk of developing acute HF in patients with COVID-19 during hospital admission. Some authors have studied the incidence of acute heart failure in patients with COVID-19 infection. Berg et al. reported that 8.9% of the patients had acute HF, including 12.0% with classic cardiogenic shock and 34.1% with vasodilatory CS. The majority were de novo HF presentations [15]. This incidence ranged from 9% to 11% in patients admitted to intensive care unit [15]. Rey et al. reported that acute heart failure was more frequently developed in patients with a previous history of CHF (11.2% vs. 2.1%; *p*  <  0.001) and had higher levels of N-terminal pro brain natriuretic peptide. Arrhythmias and CHF were the main predictors of the development of AHF. In their study, these subsets of patients (CHF) had higher mortality rates (48.7% vs. 19.0%; *p*  <  0.001) as well as those developing AHF (46.8% vs. 19.7%; *p*  <  0.001) [16]. In our study, we reported 5.8%, probably because we included in the analysis many patients admitted to hospitals for COVID-19 infection who did not have risk factors for the development of HF.

In our study, we observed that patients who develop HF during hospitalization for COVID-19 infection were older and had more cardiovascular risk factors and medical comorbidities than those who did not develop this complication. These findings are compatible because it has been observed that patients with HF are very often older patients with cardiovascular risk factors such as hypertension and diabetes, previous diseases such as ischemic heart disease, atrial fibrillation, or chronic renal failure [17].

Coronary heart disease, diabetes, obesity, and hypertension are risk factors that usually precede the development of HF [17,18]. In our study, indeed, the above risk factors were much more prevalent among those who developed HF during admission compared with those who did not develop this complication. The association of the development of HF with other diseases such as COPD, HIV, dementia, or solid cancer without metastasis, we believe, would need further study to ensure that these could be independent factors for the development of the disease in these situations.

The differences found in terms of symptoms in the different groups, such as ageusia, anosmia, and neurological symptoms, have not been reported in other studies analyzing patients with COVID-19 and HF. We should point out that dyspnea was much more frequent in the group of patients who developed acute HF, and that those subjects who presented symptoms of anosmia, ageusia, and arthromyalgias developed less dyspnea as already reported by Rubio-Rivas M et al., these symptoms were associated with a better prognosis [19]. This may be because these latter clinical manifestations tend to be more frequent in younger subjects with a lower risk of developing HF.

Regarding the analytical determinations, patients who developed acute HF as a complication during admission had higher creatinine values than patients without acute HF. The development of renal failure was also associated with a higher frequency of acute HF in this regard [20]. The presence of stress hyperglycemia or previous diabetes was also higher in the group of patients with HF. We know that patients with diabetes have a higher risk of being admitted and developing acute HF than patients without diabetes [20].

It has been shown that the use of ACE inhibitors reduces morbidity and mortality in patients with HF and also prevents the development of HF in patients with asymptomatic ventricular dysfunction [18]. Although regular treatment with ACE inhibitors appears to be a factor associated with the development of HF, in this case, it may be acting as a confounding factor because it is a common treatment for patients with previous HF, nephropathy, or hypertension, which are risk factors for HF. This fact observed with the ACE inhibitors can also be transferred to other treatments commonly used in HF and cardiovascular disease, such as statins or anticoagulant drugs [21]. Casas Rojo et al. showed a protective role of ACEI/ARA2 and statins [14].

Treatment during hospitalization with various antibiotics (beta-lactams or quinolones), corticosteroids, and high-flow oxygen nasal therapy confer a greater risk for the development of HF, which could be due to the fact that they actually favored the development of this complication or, more plausibly, to the fact that these treatments were given to patients who developed an infection as a complication in the case of antibiotics or who were in a worse condition in the case of corticosteroids and nasal oxygen supply [22,23]. In the SEMI-COVID-19 registry, the use of corticosteroids and treatment with antibiotics was associated with a worse prognosis in patients with HF [19].

In the multivariate analysis of the predictors of the development of HF, age, atrial fibrillation, arterial hypertension, recent myocardial infarction, and the Charlson index were found to be independent predictors of the development of this complication. Other factors such as dyslipidemia, diabetes, and renal failure, which in the bivariate analysis appear to be associated with statistical significance to the development of HF, were found not to be predictors in the multivariate analysis and could be confounded due to their association with the previous variables. Nevertheless, this paradoxical finding requires further studies to confirm due to the limitations of this study.

The fact that atrial fibrillation appears as a predictor for the development of HF is consistent with findings found in other studies in which patients with atrial fibrillation had higher rates of mortality and complications [24]. In another study, it was observed that 5% of patients hospitalized for COVID-19 developed a first episode of atrial fibrillation and had higher rates of hypertension and HF than those who did not develop atrial fibrillation [25]. In this same study, arterial hypertension and age are factors related to the severity of the infection, while diabetes and sex do not have a statistically significant relationship with it [25]. In our study, 39.1% of HF patients with COVID-19 presented atrial fibrillation. The systematic review and meta-analysis conducted by Romiti et al. [26] reported a prevalence of 8% (95% CI: 6.3–10.2%, 95%: 2.0–27.1%) but it must be highlighted that they included AF in all COVID-19 patients, and we do not know the prevalence of AF in the group of HF patients in this study. However, they reported an association between AF and chronic heart failure in COVID-19 patients. Furthermore, AF COVID-19 patients in that study were less likely to be female in contrast to our results. Our prevalence could also be explained by the fact that patients were much older compared to those in that analysis in which mean age ranges from 50 to 68 years old [26].

Patients admitted for COVID-19 complicated by the development of HF during admission present a higher rate of in-hospital mortality and more complications such as the development of respiratory failure, sepsis, shock, admission to the ICU, and the need for mechanical ventilation.

It has been shown that those patients admitted with a history of HF and COVID-19 have up to twice the mortality, as well as a more severe and prolonged clinical course of the disease, a difference that persists after adjusting for the various variables, which points to previous heart failure as an independent factor of morbidity and mortality in COVID-19 [1,27]. The presence of both de novo and exacerbated HF in chronic patients is associated with a 50% mortality during admission [19].

This study has some limitations: (1) It is a cross-sectional, retrospective study, which prevents establishing cause–effect relationships and can lead to confounding factors as explained above for the usual treatments for HF. (2) Although HF has a clinical definition and diagnosis, this diagnosis is usually supported in clinical practice by complementary tests such as echocardiography and the analytical determination of NT-pro-BNP. We have not been able to obtain results for both parameters, which reduces the objectivity of the diagnosis of HF. We were not able to obtain echocardiographic data regarding right ventricular dysfunction (RVD) that may have been relevant as prognostic significance. In this sense, Corica et al. reported that the presence of RVD had been associated with an increased likelihood of all-cause death (OR 3.32, 95% CI 1.94–5.70). Furthermore, RVD was found in 1 out of 5 COVID-19 patients and was associated with all-cause mortality [28]. (3) We were unable to analyze the time-to-event data. There is a clear high risk of competing events which is not accounted for here. If a patient died before developing HF, he/she could not be at risk of developing HF as well. This is important when trying to identify predictors of HF, as those more associated with both risk of mortality and HF may not show a significant relationship with HF due to the risk of competing events. (4) Data were analyzed according to a logistic regression model.

## 5. Conclusions

Patients who presented a higher risk of developing HF were older with cardiovascular risk factors. The risk factors for HF development were age, atrial fibrillation, obesity, and peripheral vascular disease. In addition, patients who developed HF more frequently required to be intubated or admitted to the ICU.

## Figures and Tables

**Table 1:** Epidemiologic variables and medical history of patients with COVID-19 with and without acute heart failure.

Variable	Total (*n* = 16,474)	HF (*n* = 958)	No HF (*n* = 15,516)	*p*-Value
Age (years, SD)	67 ± 12	79 ± 12.04	66.63 ± 16.05	<0.001
Sex (Women) (N,%)	7008 (42.5%)	420 (43.8%)	6588 (42.5%)	0.401
Comorbidities				
Obesity (N,%)	3347 (22.1%)	253 (28.8%)	3094 (21.6%)	<0.001
Hypertension (N,%)	8437 (51.3%)	733 (76.7%)	7704 (49.7%)	<0.001
Diabetes without organ damage (N,%)	2392 (14.5%)	187 (19.6%)	2205 (14.2%)	<0.001
Diabetes with organ damage (N,%)	887 (5.4%)	136 (14.2%)	751 (4.8%)	<0.001
Dyslipemia (N,%)	6493 (39.5%)	491 (51.3%)	6002 (38.7%)	<0.001
Atrial fibrillation (N,%)	1822 (11.1%)	375 (39.1%)	1447 (9.3%)	<0.001
Angor (N,%)	576 (3.5%)	88 (9.2%)	488 (3.1%)	<0.001
Myocardial infarction (N,%)	934 (5.7%)	149 (15.6%)	785 (5.1%)	<0.001
Heart failure (N/%)	1168 (7.1%)	389 (40.6%)	779 (5.0%)	<0.001
Peripheral vascular disease (N,%)	753 (4.6%)	114 (11.9%)	639 (4.1%)	<0.001
COPD (N,%)	1123 (6.8%)	152 (15.9%)	971 (6.3%)	<0.001
Solid cancer without metastasis (N,%)	1022 (6.2%)	91 (9.5%)	931 (6.0%)	<0.001
Solid cancer without metastasis (N,%)	321 (2.0%)	23 (2.4%)	298 (1.9%)	0.296
Dementia (N,%)	1614 (9.8%)	166 (17.3%)	1448 (9.3%)	<0.001

Legend: HF, heart failure; BMI, body mass index; COPD, chronic obstructive pulmonary disease.

**Table 2:** Symptoms and signs in patients with COVID-19 with and without acute heart failure.

Variable	Total (*n* = 16,474)	HF (*n* = 958)	No HF (*n* = 15,516)	*p*-Value
Anorexia (N,%)	3233 (19.9%)	224 (23.8%)	3009 (19.7%)	0.002
Fatigue (N,%)	7072 (43.4%)	529 (44.1%)	6655 (43.4%)	0.675
Ageusia (N,%)	1373 (8.5%)	30 (3.2%)	1343 (89%)	<0.001
Anosmia (N,%)	1221 (7.6%)	28 (3.0%)	1193 (7.9%)	<0.001
Flu-like symptoms				
Headache (N,%)	1959 (12.0%)	77 (8.2%)	1882 (12.3%)	<0.001
Arthromyalgias (N,%)	5013 (30.7%)	213 (22.5%)	4800 (31.2%)	<0.001
Sore throat (N,%)	1603 (9.9%)	71 (7.5%)	1532 (10.0%)	0.013
Gastrointestinal symptoms				
Vomiting (N,%)	1277 (7.8%)	58 (6.1%)	1219 (7.9%)	0.043
Diarrhea (N,%)	3939 (24.1%)	166 (17.5%)	3773 (24.5%)	<0.001
Nausea (N,%)	2002 (12.3%)	74 (7.9%)	1928 (12.6%)	<0.001
Neurologic symptoms				
Confusion (N,%)	1926 (11.8%)	230 (24.2%)	1696 (11.0%)	<0.001
Seizures (N,%)	111 (0.7%)	19 (2.0%)	92 (0.6%)	<0.001
Respiratory symptoms				
Dypsnea (N,%)	9477 (57.7%)	726 (75.9%)	8751 (56.6%)	<0.001
Clinical findings				
SBP (mean)	128.66	129.81	128.66	0.111
DBP (mean)	73.78	71	74	<0.001
Crackling (N,%)	8688 (53.9%)	608 (64.6%)	8080 (53.2%)	<0.001
Ronchi (N,%)	1779 (11.0%)	206 (22.0%)	1573 (10.4%)	<0.001
Wheezing (N,%)	998 (6.2%)	126 (13.4%)	872 (5.7%)	<0.001

Legend: HF, heart failure; SBP, systolic blood pressure; DBP, diastolic blood pressure.

**Table 3:** Admission and usual treatments of patients with COVID-19 with and without acute heart failure.

Variable	Total (*n* = 16,474)	HF (*n* = 958)	No HF (*n* = 15,516)	*p*-Value
Cardiovascular treatment				
ACE inhibitors (N,%)	2895 (17.6%)	220 (23.1%)	2675 (17.3%)	<0.001
ARABs antagonists (N,%)	3152 (19.2%)	264 (27.7%)	2888 (18.7%)	<0.001
Statins (N,%)	5265 (32.1%)	412 (43.2%)	4853 (31.4%)	<0.001
Aspirin (N,%)	2564 (15.6%)	242 (25.5%)	2322 (15.0%)	<0.001
Vitamin K antagonists (N,%)	1010 (6.2%)	203 (21.3%)	807 (5.2%)	<0.001
DOACs	712 (4.3%)	134 (16. 7%)	578 (3.7%)	<0.001
Low molecular weight heparin (N,%)	120 (0.7%)	20 (2.1%)	100 (0.6%)	<0.001
GLP-1 receptor agonists (N,%)	201 (1.2%)	18 (1.9%)	183 (1.2%)	0.056
DPP-4 inhibitors (N,%)	1122 (6.9%)	122 (12.9%)	1000 (6.5%)	<0.001
SGLT2 inhibitors (N,%)	441 (2.7%)	34 (3.6%)	407 (2.6%)	0.085
Colchicine (N,%)	140 (0.9%)	18 (1.9%)	122 (0.8%)	<0.001
Treatment during hospital admission				
Antibiotics				
Beta-lactamics (N,%)	11,648 (71.0%)	750 (78.6%)	10,898 (70.5%)	10,898 (70.5)
Macrolides (N,%)	9777 (59.7%)	523 (55%)	9254 (59.9%)	0.003
Quinolones (N,%)	2147 (13.2%)	170 (18%)	1977 (12.9%)	<0.001
Systemic steroids (N,%)	725 (4.4%)	74 (7.7%)	651 (4.2%)	<0.001
Other treatments				
Hydroxychloroquine (N,%)	85 (0.5%)	7 (0. 7%)	78 (0.5%)	0.339
Tocilizumab (N,%)	1459 (8.9%)	98 (10.3%)	1361 (8.8%)	0.117
High-flow nasal oxygen (N,%)	1396 (8.5%)	123 (13%)	1273 (8.3%)	<0.001

**Legend:** GLP-1, Glucagon-like peptide-1; DPP-4, Dipeptidyl peptidase-4 ; SGLT2, sodium-glucose cotransporter 2. ACE, angiotensin-converting enzyme inhibitor; ARABs, Angiotensin II receptor blockers (ARBs); DOACs, directly acting oral anticoagulants; GLP-1, Glucagon-like peptide-1; DPP-4, Dipeptidyl peptidase-4; SGLT2, sodium-glucose cotransporter 2.

**Table 4:** Complications during hospital admission.

Variable	Total (*n* = 16,474)	HF (*n* = 958)	No HF (*n* = 15,516)	*p*-Value
Pneumonía (N,%)	1818 (11.0%)	246(25,8%)	1572 (10.1%)	<0.001
Miocarditis (N,%)	157 (1.0%)	44 (4.6%)	113 (0.7%)	<0.001
Acute renal failure (N,%)	2256 (13.7%)	408 (42.6%)	1848 (11.9%)	<0.001
Multiorgan failure (N,%)	979 (6.0%)	223 (23.4%)	756 (4.9%)	<0.001
Prono position (N,%)	1799 (11.0%)	174 (18.2%)	1625 (10.5%)	<0.001
Non-invasive mechanical ventilation (N,%)	866 (5.3%)	104 (10.9%)	762 (4.9%)	<0.001
Sepsis (N,%)	1038 (6.3%)	188 (19.6%)	850 (5.5%)	<0.001
Intravascular disseminated disease(N,%)	178 (1.1%)	34 (3.6%)	144 (0.9%)	<0.001
Shock (N,%)	751 (4.6%)	140 (14.7%)	611(3.9%)	<0.001
ICU admission (N,%)	1464 (8.9%)	156 (16.3%)	1308 (8.4%)	<0.001
Readmission (N,%)	590 (3.6%)	54 (5.8%)	536 (3.5%)	<0.001
Mortality	3437 (21%)	511 (54.1%)	2926 (19.1%)	<0.001

**Legend:** ICU, intensive care unit.

**Table 5:** Multivariate analysis of predictors for the development of heart failure based on age variables and associated comorbidities assessed at admission.

Variables	OR	95% CI	*p*-Value
Age	1.042	1.035–1.050	<0.001
Hypertension	1.186	0.986–1.426	0.071
Heart failure	5.649	4.726–6.753	0.004
Dyslipemia	0.909	0.794–1.091	0.376
Atrial fibrillation	2.022	1.697–2.410	<0.001
Obesity (BMI ≥ 30 kg/m^2^)	1.460	1.230–1.733	<0.001
Myocardial infarction	1.358	1.077–1.739	0.010
Dementia	0.861	0.695–1.067	0.172
Diabetes without organ damage	1.204	0.994–1.458	0.058
COPD	1.405	1.127–1.752	0.002
Peripheral vascular disease	1.564	1.217–2.201	<0.001

**Legend:** BMI, body mass index; COPD, chronic obstructive pulmonary disease.

## Data Availability

The data presented in this study are available on request from the corresponding author. The data are not publicly available due to privacy restrictions.

## Supplementary Materials

The following supporting information can be downloaded at: https://www.mdpi.com/article/10.3390/jcm12144649/s1, Table S1: Analytical data of patients with COVID-19 with and without acute heart failure.

## Appendix A. List of the SEMI-COVID-19 Network members

**Coordinator of the SEMI-COVID-19 Registry**: José Manuel Casas Rojo.

**Members of the SEMI-COVID-19 Group**:

Hospital. Universitario de Bellvitge. L’Hospitalet de Llobregat (Barcelona): Xavier Corbella, Francesc Formiga Pérez, Narcís Homs, Abelardo Montero, Jose María Mora-Luján, Manuel Rubio-Rivas.

Hospital. Universitario 12 de Octubre. (Madrid): Paloma Agudo de Blas, Coral Arévalo Cañas, Blanca Ayuso, José Bascuñana Morejón, Samara Campos Escudero, María Carnevali Frías, Santiago Cossio Tejido, Borja de Miguel Campo, Carmen Díaz Pedroche, Raquel Diaz Simon, Ana García Reyne, Laura Ibarra Veganzones, Lucia Jorge Huerta, Antonio Lalueza Blanco, Jaime Laureiro Gonzalo, Jaime Lora-Tamayo, Carlos Lumbreras Bermejo, Guillermo Maestro de la Calle, Rodrigo Miranda Godoy, Barbara Otero Perpiña, Diana Paredes Ruiz, Marcos Sánchez Fernández, Javier Tejada Montes.

Hospital Costa del Sol. Marbella (Málaga): Victoria Augustín Bandera, Javier García Alegría, Nicolás Jiménez-García, Jairo Luque del Pino, María Dolores Martín Escalante, Francisco Navarro Romero, Victoria Nuñez Rodriguez, Julián Olalla Sierra.

Hospital Universitario Gregorio Marañon. (Madrid): Laura Abarca Casas, Álvaro Alejandre de Oña, Rubén Alonso Beato, Leyre Alonso Gonzalo, Jaime Alonso Muñoz, Crhistian Mario Amodeo Oblitas, Cristina Ausín García, Marta Bacete Cebrián, Jesús Baltasar Corral, Maria Barrientos Guerrero, Alejandro D. Bendala Estrada, María Calderón Moreno, Paula Carrascosa Fernández, Raquel Carrillo, Sabela Castañeda Pérez, Eva Cervilla Muñoz, Agustín Diego Chacón Moreno, Maria Carmen Cuenca Carvajal, Sergio de Santos, Andrés Enríquez Gómez, Eduardo Fernández Carracedo, María Mercedes Ferreiro-Mazón Jenaro, Francisco Galeano Valle, Alejandra Garcia, Irene Garcia Fernandez-Bravo, María Eugenia García Leoni, María Gómez Antúnez, Candela González San Narciso, Anthony Alexander Gurjian, Lorena Jiménez Ibáñez, Cristina Lavilla Olleros, Cristina Llamazares Mendo, Sara Luis García, Víctor Mato Jimeno, Clara Millán Nohales, Jesús Millán Núñez-Cortés, Sergio Moragón Ledesma, Antonio Muiño Míguez, Cecilia Muñoz Delgado, Lucía Ordieres Ortega, Susana Pardo Sánchez, Alejandro Parra Virto, María Teresa Pérez Sanz, Blanca Pinilla Llorente, Sandra Piqueras Ruiz, Guillermo Soria Fernández-Llamazares, María Toledano Macías, Neera Toledo Samaniego, Ana Torres do Rego, Maria Victoria Villalba Garcia, Gracia Villarreal, María Zurita Etayo.

Hospital de Cabueñes. Gijón (Asturias): Ana María Álvarez Suárez, Carlos Delgado Vergés, Rosa Fernandez-Madera Martínez, Eva Mª Fonseca Aizpuru, Alejandro Gómez Carrasco, Cristina Helguera Amezua, Juan Francisco López Caleya, Diego López Martínez, María del Mar Martínez López, Aleida Martínez Zapico, Carmen Olabuenaga Iscar, Lucía Pérez Casado, María Luisa Taboada Martínez, Lara María Tamargo Chamorro.

Hospital Regional Universitario de Málaga: Mª Mar Ayala-Gutiérrez, Rosa Bernal López, José Bueno Fonseca, Verónica Andrea Buonaiuto, Luis Francisco Caballero Martínez, Lidia Cobos Palacios, Clara Costo Muriel, Francis de Windt, Ana Teresa Fernandez-Truchaud Christophel, Paula García Ocaña, Ricardo Gómez Huelgas, Javier Gorospe García, José Antonio Hurtado Oliver, Sergio Jansen-Chaparro, Maria Dolores López-Carmona, Pablo López Quirantes, Almudena López Sampalo, Elizabeth Lorenzo-Hernández, Juan José Mancebo Sevilla, Jesica Martín Carmona, Luis Miguel Pérez-Belmonte, Iván Pérez de Pedro, Araceli Pineda-Cantero, Carlos Romero Gómez, Michele Ricci, Jaime Sanz Cánovas.

Hospital Universitario La Paz. (Madrid): Jorge Álvarez Troncoso, Francisco Arnalich Fernández, Francisco Blanco Quintana, Carmen Busca Arenzana, Sergio Carrasco Molina, Aranzazu Castellano Candalija, Germán Daroca Bengoa, Alejandro de Gea Grela, Alicia de Lorenzo Hernández, Alejandro Díez Vidal, Carmen Fernández Capitán, Maria Francisca García Iglesias, Borja González Muñoz, Carmen Rosario Herrero Gil, Juan María Herrero Martínez, Víctor Hontañón, Maria Jesús Jaras Hernández, Carlos Lahoz, Cristina Marcelo Calvo, Juan Carlos Martín Gutiérrez, Monica Martinez Prieto, Elena Martínez Robles, Araceli Menéndez Saldaña, Alberto Moreno Fernández, Jose Maria Mostaza Prieto, Ana Noblejas Mozo, Carlos Manuel Oñoro López, Esmeralda Palmier Peláez, Marina Palomar Pampyn, Maria Angustias Quesada Simón, Juan Carlos Ramos Ramos, Luis Ramos Ruperto, Aquilino Sánchez Purificación, Teresa Sancho Bueso, Raquel Sorriguieta Torre, Clara Itziar Soto Abanedes, Yeray Untoria Tabares, Marta Varas Mayoral, Julia Vásquez Manau.

Hospital Royo Villanova. (Zaragoza): Nicolás Alcalá Rivera, Anxela Crestelo Vieitez, Esther del Corral Beamonte, Jesús Díez Manglano, Isabel Fiteni Mera, Maria del Mar Garcia Andreu, Martin Gericó Aseguinolaza, Cristina Gallego Lezaun, Claudia Josa Laorden, Raul Martínez Murgui, Marta Teresa Matía Sanz.

Hospital. Universitario Reina Sofía. (Córdoba): Antonio Pablo Arenas de Larriva, Pilar Calero Espinal, Javier Delgado Lista, Francisco Fuentes-Jiménez, María del Carmen Guerrero Martínez, María Jesús Gómez Vázquez, Jose Jiménez Torres, Laura Limia Pérez, José López-Miranda, Laura Martín Piedra, Marta Millán Orge, Javier Pascual Vinagre, Pablo Pérez-Martinez, María Elena Revelles Vílchez, Angela Rodrigo Martínez, Juan Luis Romero Cabrera, José David Torres-Peña.

Hospital Clínico de Santiago de Compostela (A Coruña): Maria del Carmen Beceiro Abad, Maria Aurora Freire Romero, Sonia Molinos Castro, Emilio Manuel Paez Guillan, María Pazo Nuñez, Paula Maria Pesqueira Fontan.

Hospital Universitario Puerta de Hierro. (Madrid): Ane Andrés Eisenhofer, Ana Arias Milla, Isolina Baños Pérez, Laura Benítez Gutiérrez, Javier Bilbao Garay, Jorge Calderón Parra, Alejandro Callejas Díaz, Erika Camacho Da Silva, M ª Cruz Carreño Hernández, Raquel Castejón Díaz, María Jesús Citores Sánchez, Carmen Cubero Gozalo, Valentín Cuervas-Mons Martínez, Laura Dorado Doblado, Sara de la Fuente Moral, Alberto Díaz de Santiago, Itziar Diego Yagüe, Ignacio Donate Velasco, Ana María Duca, Pedro Durán del Campo, Gabriela Escudero López, Esther Expósito Palomo, Ana Fernández Cruz, Amy Galán Gómez, Sonia García Prieto, Beatriz García Revilla, Miguel Ángel García Viejo, Javier Gómez Irusta, Patricia González Merino, Edith Vanessa Gutiérrez Abreu, Isabel Gutiérrez Martín, Ángela Gutiérrez Rojas, Andrea Gutiérrez Villanueva, Jesús Herráiz Jiménez, Fátima Ibáñez Estéllez, Pedro Laguna del Estal, Mª Carmen Máinez Sáiz, Carmen de Mendoza Fernández, María Martínez Urbistondo, Fernando Martínez Vera, María Mateos Seirul-lo, Susana Mellor Pita, Patricia A. Mills Sánchez, Esther Montero Hernández, Alberto Mora Vargas, Victor Moreno-Torres Concha, Ignacio Morrás De La Torre, Elena Múñez Rubio, Rosa Muñoz de Benito, Alejandro Muñoz Serrano, Pablo Navarro Palomo, Ilduara Pintos Pascual, Arturo José Ramos Martín-Vegue, Antonio Ramos Martínez, Celia Rodríguez Olleros, Alberto Roldán Montaud, Yolanda Romero Pizarro, Silvia Rosado García, Diana Ruiz de Domingo, David Sánchez Ortiz, Enrique Sánchez Chica, Irene Solano Almena, Elena Suanzes Martin, Yale Tung Chen, Pablo Tutor de Ureta, Ángela Valencia Alijo, Jose Manuel Vázquez Comendador, Juan Antonio Vargas Núñez.

Hospital Universitario Dr. Peset. (Valencia): Juan Alberto Aguilera Ayllón, Arturo Artero, María del Mar Carmona Martín, María José Fabiá Valls, Maria de Mar Fernández Garcés, Ana Belén Gómez Belda, Ian López Cruz, Manuel Madrazo López, Elisabeth Mateo Sanchis, Jaume Micó Gandia, Laura Piles Roger, Adela Maria Pina Belmonte, Alba Viana García.

Hospital Clínico San Carlos. Madrid: Inés Armenteros Yeguas, Javier Azaña Gómez, Julia Barrado Cuchillo, Irene Burruezo López, Noemí Cabello Clotet, Alberto E. Calvo Elías, Elpidio Calvo Manuel, Carmen María Cano de Luque, Cynthia Chocron Benbunan, Laura Dans Vilan, Claudia Dorta Hernández, Ester Emilia Dubon Peralta, Vicente Estrada Pérez, Santiago Fernandez-Castelao, Marcos Oliver Fragiel Saavedra, José Luis García Klepzig, Maria del Rosario Iguarán Bermúdez, Esther Jaén Ferrer, Alejandro Maceín Rodríguez, Alejandro Marcelles de Pedro, Rubén Ángel Martín Sánchez, Manuel Méndez Bailón, Sara Miguel Álvarez, Maria José Nuñez Orantos, Carolina Olmos Mata, Eva Orviz García, David Oteo Mata, Cristina Outon González, Juncal Perez-Somarriba, Pablo Pérez Mateos, Maria Esther Ramos Muñoz, Xabier Rivas Regaira, Laura Mª Rodríguez Gallardo, Iñigo Sagastagoitia Fornie, Alejandro Salinas Botrán, Miguel Suárez Robles, Maddalena Elena Urbano, Andrea María Vellisca González, Miguel Villar Martínez.

Hospital Universitario de Badajoz: Rafael Aragon Lara, Inmaculada Cimadevilla Fernandez, Juan Carlos Cira García, Gema Maria García García, Julia Gonzalez Granados, Beatriz Guerrero Sánchez, Francisco Javier Monreal Periáñez, Maria Josefa Pascual Perez.

Hospital Universitario San Juan de Alicante (Alicante): Marisa Asensio Tomás, David Balaz, David Bonet Tur, Ruth Cañizares Navarro, Paloma Chazarra Pérez, Jesús Corbacho Redondo, Eliana Damonte White, María Escamilla Espínola, Leticia Espinosa Del Barrio, Pedro Jesús Esteve Atiénzar, Carles García Cervera, David Francisco García Núñez, Francisco Garrido Navarro, Vicente Giner Galvañ, Angie Gómez Uranga, Javier Guzmán Martínez, Isidro Hernández Isasi, Lourdes Lajara Villar, Verónica Martínez Sempere, Juan Manuel Núñez Cruz, Sergio Palacios Fernández, Juan Jorge Peris García, Rafael Piñol Pleguezuelos, Andrea Riaño Pérez, José Miguel Seguí Ripoll, Azucena Sempere Mira, Philip Wikman-Jorgensen.

Hospital Universitario de Elda (Alicante): Carmen Cortés Saavedra, Jennifer Fernández Gómez, Borja González López, María Soledad Hernández Garrido, Ana Isabel López Amorós, Santiago López Gil, Maria de los Reyes Pascual Pérez, Nuria Ramírez Perea, Andrea Torregrosa García.

Hospital Universitario Infanta Cristina. Parla (Madrid): Juan Miguel Antón Santos, Ana Belén Barbero Barrera, Blanca Beamonte Vela, Coralia Bueno Muiño, Charo Burón Fernández, Ruth Calderón Hernáiz, Irene Casado López, José Manuel Casas Rojo, Andrés Cortés Troncoso, Pilar Cubo Romano, Francesco Deodati, Alejandro Estrada Santiago, Gonzalo García Casasola Sánchez, Elena García Guijarro, Francisco Javier García Sánchez, Pilar García de la Torre, Mayte de Guzmán García-Monge, Davide Luordo, María Mateos González, José A. Melero Bermejo, Cruz Pastor Valverde, José Luis Pérez Quero, Fernando Roque Rojas, Lorea Roteta García, Elena Sierra Gonzalo, Francisco Javier Teigell Muñoz, Juan Vicente de la Sota, Javier Villanueva Martínez.

Hospital de Pozoblanco (Córdoba): José Nicolás Alcalá Pedrajas, Antonia Márquez García, Inés Vargas.

Hospital Santa Marina. Bilbao: María Areses Manrique, Ainara Coduras Erdozain, Ane Labirua-Iturburu Ruiz.

Hospital Universitario Son Llàtzer. (Palma de Mallorca): Andrés de la Peña Fernández, Almudena Hernández Milián.

Hospital San Pedro. Logroño (La Rioja): Diana Alegre González, Irene Ariño Pérez de Zabalza, Sergio Arnedo Hernández, Jorge Collado Sáenz, Beatriz Dendariena, Marta Gómez del Mazo, Iratxe Martínez de Narvajas Urra, Sara Martínez Hernández, Estela Menendez Fernández, Jose Luís Peña Somovilla, Elisa Rabadán Pejenaute.

Hospital de Mataró. (Barcelona): Raquel Aranega González, Ramon Boixeda, Javier Fernández Fernández, Carlos Lopera Mármol, Marta Parra Navarro, Ainhoa Rex Guzmán, Aleix Serrallonga Fustier.

Hospital Universitario de Ferrol (A Coruña): Hortensia Alvarez Diaz, Tamara Dalama Lopez, Estefania Martul Pego, Carmen Mella Pérez, Ana Pazos Ferro, Sabela Sánchez Trigo, Dolores Suarez Sambade, Maria Trigas Ferrin, Maria del Carmen Vázquez Friol, Laura Vilariño Maneiro.

Hospital Infanta Margarita. Cabra (Córdoba): María Esther Guisado Espartero, Lorena Montero Rivas, Maria de la Sierra Navas Alcántara, Raimundo Tirado-Miranda.

Hospital Universitario de Salamanca: Gloria María Alonso Claudio, Víctor Barreales Rodríguez, Cristina Carbonell Muñoz, Adela Carpio Pérez, María Victoria Coral Orbes, Daniel Encinas Sánchez, Sandra Inés Revuelta, Miguel Marcos Martín, José Ignacio Martín González, José Ángel Martín Oterino, Leticia Moralejo Alonso, Sonia Peña Balbuena, María Luisa Pérez García, Ana Ramon Prados, Beatriz Rodríguez-Alonso, Ángela Romero Alegría, Maria Sanchez Ledesma, Rosa Juana Tejera Pérez.

Hospital Universitario Virgen del Rocío. (Sevilla): Reyes Aparicio Santos, Máximo Bernabeu-Wittel, Santiago Rodríguez Suárez, María Nieto, Luis Giménez Miranda, Rosa María Gámez Mancera, Fátima Espinosa Torre, Carlos Hernandez Quiles, Concepción Conde Guzmán, Juan Delgado de la Cuesta, Jara Eloisa Ternero Vega, María del Carmen López Ríos, Pablo Díaz Jiménez, Bosco Baron Franco, Carlos Jiménez de Juan, Sonia Gutiérrez Rivero, Julia Lanseros Tenllado, Verónica Alfaro Lara, Aurora González Estrada.

Hospital Público de Monforte de Lemos (Lugo): José López Castro, Manuel Lorenzo López Reboiro, Cristina Sardiña González.

Hospital Marina Baixa. Villajoyosa (Alicante): Javier Ena, José Enrique Gómez Segado.

Hospital General Defensa. (Zaragoza): Anyuli Gracia Gutiérrez, Leticia Esther Royo Trallero.

Hospital Universitario Quironsalud (Madrid): Pablo Guisado Vasco, Ana Roda Santacruz, Ana Valverde Muñoz.

Hospital Comarcal de Blanes (Girona): Oriol Alonso Gisbert, Mercé Blázquez Llistosella, Pere Comas Casanova, Angels Garcia Flores, Anna Garcia Hinojo, Ana Inés Méndez Martínez, Maria del Carmen Nogales Nieves, Agnés Rivera Austrui, Alberto Zamora Cervantes.

Hospital do Salnes. Vilagarcía de Arousa (Pontevedra): Vanesa Alende Castro, Ana María Baz Lomba, Ruth Brea Aparicio, Marta Fernández Morales, Jesús Manuel Fernández Villar, María Teresa López Monteagudo, Cristina Pérez García, Lorena Rodríguez Ferreira, Diana Sande Llovo, Maria Begoña Valle Feijoo.
