# Supplementary material for: Prognostic Factors Associated with Acute Heart Failure in Patients Admitted for COVID-19: Analysis of the SEMI-COVID-19 Registry
Source: J Clin Med. 2023 Jul 12;12(14):4649. doi: 10.3390/jcm12144649 (PMC10380653; doi:10.3390/jcm12144649)
Supplement: Supplementary file 1 [file jcm-12-04649-s001.zip › jcm-2415867-supplementary.pdf]

**Supplementary table S1.** Analytical data of patients with COVID-19 with and without acute heart failure.

| Variable | Total (n=16474) | HF (n=958) | No HF (n=15516) | p-value |
|---|---|---|---|---|
| O2 saturation (mean, SD) | 92±5.9 | 90.6±7.2 | 93±5.8 | <0.001 |
| pH at admission (mean, SD) | 7.43±0.19 | 7.41±0. 003 | 7.43±0.002 | 0.049 |
| Hemoglobin (g/dL) (mean, SD) | 13.6±1.89 | 12.6±2.3 | 13.7±1.8 | <0.001 |
| Platelets (mean, SD) | 207.395±935 | 197.983±324 | 207.705±749 | 0.002 |
| Leukocytes (x $10^9$/L) (mean, SD) | 7384 ±5463 | 8736±7712 | 7315±5283 | <0.001 |
| Lymphocytes (x $10^9$/L) (mean, SD) | 1061.9±2144 | 1455±4857 | 1139±1851 | <0.001 |
| Neutrophils (x $10^9$/L) (mean, SD) | 5532±4512 | 6637±6925 | 5485±4312 | <0.001 |
| PCR (mg/L) (mean, SD) | 88.32±89.31 | 98.6±86.9 | 88.1±89.4 | <0.001 |
| Serum creatinine (mg/dL) (mean, SD) | 1.1±0.86 | 1.43±0.93 | 1.07±0.85 | <0.001 |
| Urea (mg/dl) (mean, SD) | 48.16±37.35 | 74.52±51.16 | 46.34±35.62 | <0.001 |
| LDH (U/L) (mean, SD) | 371±219 | 427±305 | 369±213 | <0.001 |
| Total bilirubin (mmol/L) (U/L) (mean, SD) | 0.68±1.3 | 0.72±0.6 | 0.68±1.4 | 0.507 |
| Sodium (mmol/L) (mean, SD) | 137.55±4.76 | 137.9±6.12 | 137.5±4.66 | 0.017 |
| Potassium(mmol/L) (mean, SD) | 4.12±0.56 | 4.3±0.68 | 4.1±0.55 | <0.001 |
| Glycemia (mg/dl) (mmol/L) (mean, SD) | 128.34±58.96 | 145±66,27 | 127.31±58.3 | <0.001 |
| Ferritin (ug/L) (mmol/L) (mean, SD) | 946.99±1097.74 | 943±1356 | 951±1079 | 0.88 |
| IL-6 (mg/L) (mean, SD) | 70.32±204.53 | 75.9±256.06 | 70.9±220.52 | 0.783 |
| Procalcitonin (ng/mL) (mean, SD) | 0.49±2.47 | 0.78±2.15 | 0.47±2.49 | 0.004 |
| D-dimer (ng/mL) (mean, SD) | 1939±9481 | 4187±19864 | 1865±8503 | <0.001 |

Legend: HF, heart failure; PCR, protein C reactive; LDH, Lactate dehydrogenase; IL-6, interleukin 6.
